# Diode laser-based thermometry using two-line atomic fluorescence of indium and gallium

**DOI:** 10.1007/s00340-017-6855-z

**Published:** 2017-11-07

**Authors:** Jesper Borggren, Wubin Weng, Ali Hosseinnia, Per-Erik Bengtsson, Marcus Aldén, Zhongshan Li

**Affiliations:** Lund University, Lund, Sweden

## Abstract

A robust and relatively compact calibration-free thermometric technique using diode lasers two-line atomic fluorescence (TLAF) for reactive flows at atmospheric pressures is investigated. TLAF temperature measurements were conducted using indium and, for the first time, gallium atoms as temperature markers. The temperature was measured in a multi-jet burner running methane/air flames providing variable temperatures ranging from 1600 to 2000 K. Indium and gallium were found to provide a similar accuracy of ~ 2.7% and precision of ~ 1% over the measured temperature range. The reliability of the TLAF thermometry was further tested by performing simultaneous rotational CARS measurements in the same experiments.

## Introduction

Thermometric techniques that provide accurate and precise temperature measurements in reactive flows are crucial for the understanding of combustion processes, development of efficient combustion devices and improvement of kinetic modeling. Thermometric techniques providing accurate measurements with good spatial resolution and, with the ability to map the temperature field in turbulent flows are highly demanded. Several techniques have been developed for this purpose and reviews of thermometric techniques can be found in [1–3].

An established technique that has, in recent times, received increased attention from the combustion research field, as it has the possibility to provide measurements with the requirements listed above, is two-line atomic fluorescence (TLAF) [4–6]. TLAF is a laser-based ratiometric technique, in which the temperature-dependent population of two lower lying electronic states, of a seeded atom, is probed by laser excitation and the ratio of the resulting fluorescence is correlated with the temperature of the gas. TLAF offers several beneficial features for temperature measurements in reactive flows. Firstly, no a priori knowledge of the combustion environment is necessary as the technique is independent of quenching due to the excitation to a common upper state [7]. Secondly, the use of an atomic species as temperature marker offers advantages such as providing high transition probabilities yielding strong fluorescence signals as well as being free from errors related to vibrational and rotational energy transfer existing in molecules [8, 9]. Thirdly, TLAF also has the possibility for thermometry measurements in sooting and particulate-laden flames as the detection wavelength can be shifted from the excitation wavelength [10, 11]. The disadvantage of TLAF is that an atomic species, not normally present in the flame, has to be introduced which may add complexities to the experimental set-up.

Two-line atomic fluorescence has been developed in steps where the first theoretical ground work was conducted by Alkemade [7] and experimentally demonstrated shortly thereafter using atomic lamps [12, 13]. With the advent of suitable lasers, TLAF became a tool for temperature measurements with imaging capabilities in non-steady environments [4, 10, 11, 14–16]. Recently, TLAF measurement has been extended from the linear regime to non-linear regime by Medwell et al. [4] and to the saturation regime by Manteghi et al. [14] in an attempt to maximize the fluorescence signal for instantaneous temperature measurements. With the improvement in regards to available power and wavelengths of diode lasers, TLAF measurements using diode lasers have been shown to give accurate temperatures in both low-pressure and atmospheric flames [6, 17–19], although, at the expense of time-averaged measurements due to the lower laser power. The advantage of using diode lasers is that they offer better control of the excitation wavelength and more stable power output resulting in better accuracy as well as enabling simultaneous path-integrated concentration measurements.

The seeding of the atomic species is critical in TLAF and can, depending on the configuration used, adversely affect the measurement region. Traditionally, the atomic species has been introduced to the flame by dissolving a salt that contains the atomic species, e.g., InCl_3_ in the case of indium, in a liquid and thereafter use a pneumatic nebulizer to introduce metal halide particles into the flame to produce indium atoms after evaporation [20]. However, it may be difficult to accurately control the seeding concentration and the metal halide particles will not generate any free atoms until they pass through the flame. A recently introduced method of seeding indium into the flame is by laser ablation, where a laser pulse directed at an indium rod causes indium atoms and agglomerates to be released in a gas flow which, is later mixed with the flame [21]. This does, however, require a pulsed laser of sufficient strength.

The main atomic species, so far adopted in TLAF, have been indium [4, 6, 16, 22] with the exception of a few early works which used thallium [12, 13]. Thallium was later abandoned due to being temperature insensitive below 3000 K as well as having toxic properties. The atomic species used, should be sensitive in the temperature range of the experiment which is determined by the energy splitting between the two lower electronic states. Generally the energy splitting $\Delta E$ should be in the order of *kT:*

$$\Delta E\sim kT,$$

where *k* is the Boltzmann constant and *T* is the temperature to be measured. The energy splitting of indium makes it suitable as a temperature marker for flame temperatures above 1000 K [13]. There has, so far, been no investigations into temperature markers for temperatures below 1000 K, which is an important temperature range for low temperature chemistry [23]. Gallium has an energy splitting allowing for more sensitive measurements with better signal strengths at lower temperature ranges than indium and should be a suitable candidate as a temperature marker for these at lower temperatures. However, as noted in [13], the temperature precision is proportional to the reciprocal of the energy splitting resulting in a trade-off between the signal strength and the precision.

In this work, we investigate indium and, for the first time, gallium as temperature markers for two-line atomic fluorescence, in regards to accuracy and precision over temperatures ranging from 1500 K up to 2000 K. Simultaneous temperature and concentration measurements in laminar flames stabilized on a multi-jet burner [24] at atmospheric pressure. The seeding system described in [25] is used to seed the atomic species in gas phase to the flow. The system offers the possibility to seed a constant concentration of the atomic species to a wide range of burners.

## Experimental methods

**Fig. 1:**
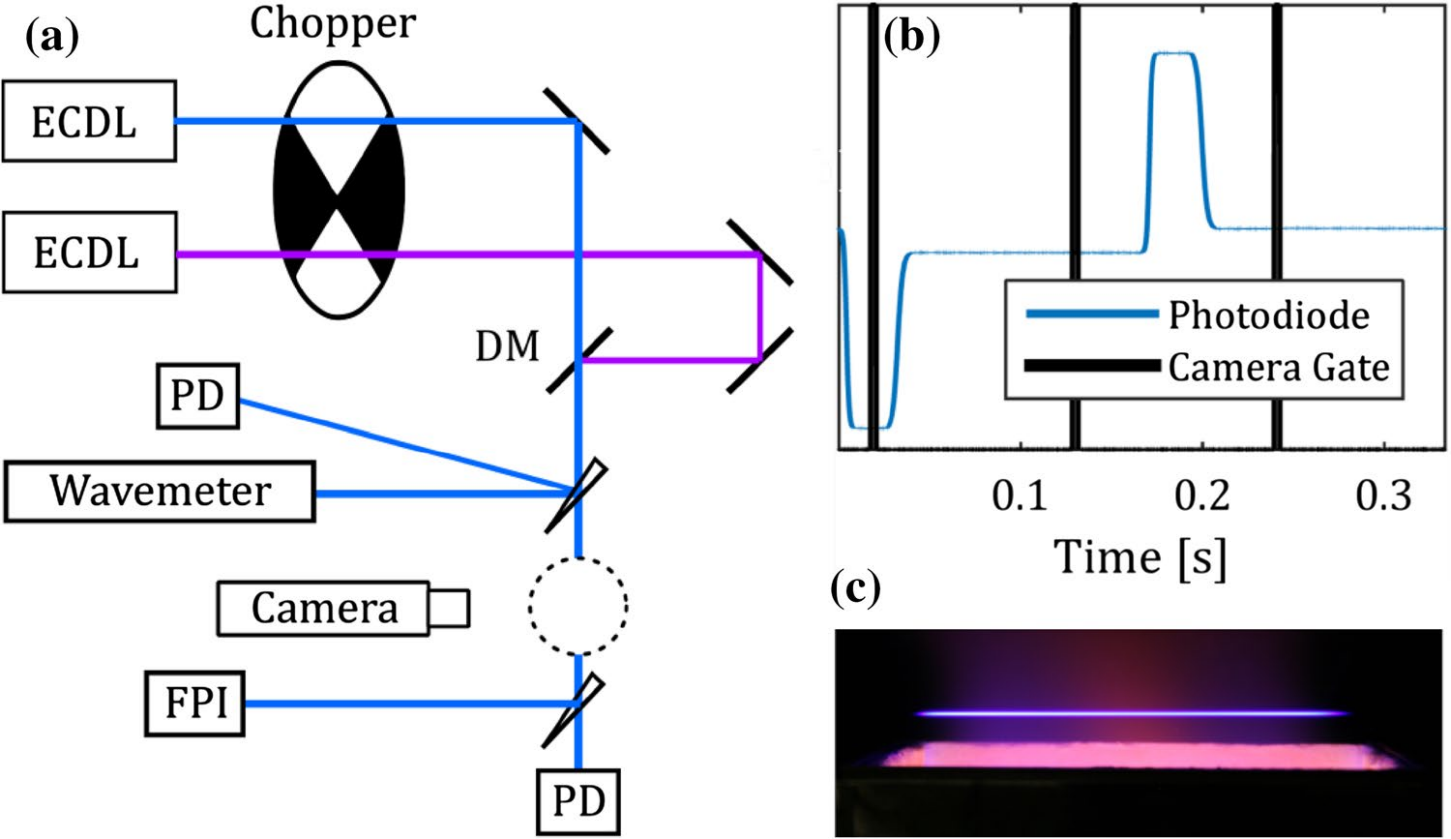
**a** Schematic of the experimental setup. *DM* dichroic mirror, *PD* photodiode, *ECDL* external cavity diode laser, *FPI* Fabry–Perot interferometer. **b** Detected laser power during a revolution of the chopper and the corresponding timing of the camera. **c** Photograph of the laser-induced fluorescence of indium in the product zone of a flame above the burner

A schematic of the optical set-up used to conduct the temperature measurements is shown in Fig. 1a; the same set-up was used for measurements with both indium and gallium with the exception of the lasers and the wavelength-dependent optics (dichroic mirror and camera filter). Two external cavity diode lasers (Toptica DL100PRO and DL100) with a laser line width < 1 MHz were used to excite the seeded atomic species. The laser power in the interrogation region was approximately 5 mW. The two laser beams were overlapped using a dichroic mirror and were left unfocused as it was observed that even a loose focus using a long focal length lens moved the LIF signal into the non-linear regime; a beam diameter of approximately 1 mm was used. Using a wedged glass plate, part of the beam was sent to a wavemeter (HighFinesse UV6-200) with a resolution of 0.1 pm to monitor the wavelength during the measurements, and another part of the beam was sent to a photodiode recording the laser power during the measurements. The wavemeter was calibrated by performing excitation scans for all four wavelengths used, i.e., two wavelengths for each atomic species as presented in Fig. 6. The photodiode was calibrated using a thermopile-based power meter (Thorlabs S302C). During measurement, the laser wavelength was set to the peak of the absorption profile of the seeded atomic species and was continuously monitored to enable possible compensation in laser drift during a measurement. The frequency stability of the lasers is in the order of minutes, much longer than the measurement time. A scanning Fabry–Perot interferometer was used to monitor the single-mode operation of the lasers. Absorption measurements for determination of the atomic concentrations were conducted with a photodiode placed after the interrogation region.

A chopper running at 3 Hz was employed to switch between the laser beams during a measurement and an intensified CCD camera with an exposure time 3 ms was used to record the fluorescence at 9 Hz as seen in Fig. 1b. This resulted in an acquisition of an image sequence of three different images during one revolution of the chopper, shown in Fig. 1b. The first image acquired the background luminescence, the second image the LIF from one laser and the third image the LIF from the other laser. This compensates for drifts in seeding concentration during a measurement. 150 images were acquired during one measurement, i.e., the background and the two LIF signals were each averaged over 50 images.

The camera was mounted with a filter detecting fluorescence only to the upper ground state, described in [18], as it requires only one detector and avoids both the need for a temperature calibration measurement and problems arising from signal trapping. However, this limits the measurements to flows without scattering particles and no elastic scattering. The influence of the Rayleigh signal was investigated by tuning the laser off the line and it was found that the diode lasers were too weak to induce any detectable Rayleigh signal. The detected LIF signal was integrated in the vertical direction to avoid complications from beam profile corrections, thus, only providing spatially resolved temperatures in the horizontal direction.

As a part of a wider effort to understand and model the combustion of biomass fuels, the temperature of a multi-jet burner was chosen to be characterized with the presented TLAF technique. The burner is designed for investigations of biomass particles and described in detail in [24]. A photograph of the burner and the laser-induced fluorescence signal is shown in Fig. 1c. The burner consists of 181 small laminar jet flames surrounded by a co-flow enclosed in an area of 6 cm by 10 cm. The burner can run a wide range of flame conditions with a large temperature range suitable in studies concerning combustion/gasification of solid fuels. A mixture of methane/nitrogen/oxygen with equivalence ratio between 0.7 and 1.3 enabled temperature measurements from 1500 to 2000 K and the equivalence range of interest to be mapped. The proportion of nitrogen to oxygen in the mixture was 1:2.5 as opposed to ratio of air which is 1:3.76. The total flow through the jets was 22 standard liters per minute (SLM) and the co-flow of nitrogen was 11 SLM. The fuel mixture was seeded with either TMIn or TMGa before entering the burner chamber.

The indium and gallium was seeded using the seeding system previously presented in [25] where an inert flow of gas passes through a bubbler, containing either trimethylindium (TMIn) or trimethylgallium (TMGa), and carries this compound to the measurement object where decomposition will produce the atoms to be probed in the measurement. The temperature of the bubbler and the flow speed through the bubbler determines the concentration of the seeded species in the flame. The seeding system has the benefit of not altering the flame properties due to the low seeded concentration while still providing a constant variable concentration of the atomic species to the flame.

## Temperature and concentration evaluation

**Fig. 2:**
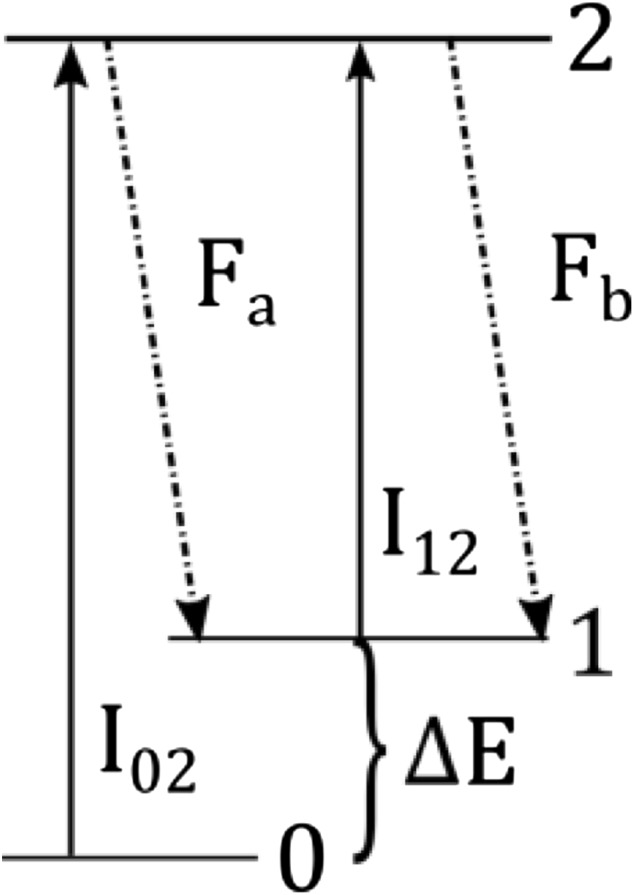
Detection scheme used for the three level system in TLAF measurements. In the current detection scheme fluorescence to the upper state (1) is detected after excitation of each state making *F*_*b*_ resonant fluorescence and sensitive to elastic scattering

In TLAF, temperature information is acquired by probing the population of the two lower lying electronic states by laser excitation and measuring the resulting fluorescence as shown in Fig. 2. From the measured fluorescence of the two states, a ratio is constructed that is correlated with the temperature of the system through the Boltzmann distribution [13, 18, 28]. The temperature in the presented technique is evaluated by comparing the measured fluorescence ratio, *R*_exp_, to a simulated ratio, *R*_sim_. For *R*_exp_ the ratio of the detected fluorescence is compensated with the laser power, as measured by the reference photodiode, and can be written as.

$$R_{ \exp } = \frac{{F_{b} /I_{12} }}{{F_{a} /I_{02} }},$$

where *F* is the detected fluorescence signal and *I* the laser power according to Fig. 2.

**Fig. 3:**
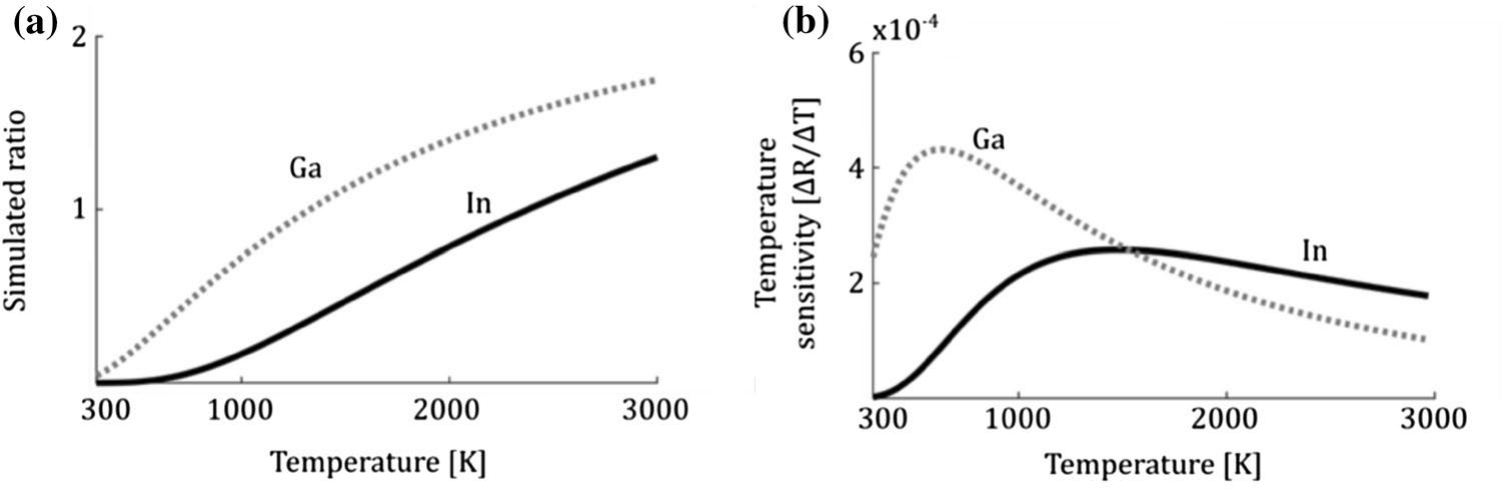
**a** Simulated fluorescence intensity ratio as a function of temperature for gallium (Ga) and indium (In) when the lasers are locked to the corresponding absorption peak. **b** Simulated temperature sensitivity of gallium and indium

To simulate the fluorescence ratio the overlap of the laser in relation to the absorption profile has to be explicitly known due to the narrow line width of the single-mode lasers which are specified to at most 1 MHz. The absorption profile for each transition is acquired from excitation scans where the wavelength is recorded on the wavemeter. The simulated ratio is, with the information of the absorption profile, calculated by simulating line shapes for the two wavelengths for temperatures between 300 and 3000 K while accounting for the laser wavelength measured during the experiment. The simulated temperature-dependent intensity ratio can be expressed as:

$$R_{\text{sim}} \left( T \right) = \frac{{g_{12} \left( {\lambda_{12} ,T} \right) \cdot f_{1} \left( T \right) \cdot B_{12} }}{{g_{02} \left( {\lambda_{02} ,T} \right) \cdot f_{0} \left( T \right) \cdot B_{02} }},$$

where $g$ is the line shape overlap as a function of wavelength, *λ*, and temperature, *T* and $f$ is the Boltzmann distribution. The Einstein coefficients for stimulated absorption, $B$, are needed to account for the probability of excitation for each transition. The simulated ratio for indium and gallium is shown in Fig. 3a for the case when the lasers are tuned to the peak of each absorption profile. The ratio of gallium is higher than indium, i.e., gallium has a higher population of the upper state at all temperatures as a result of the smaller energy splitting between the probed states for gallium. It is advantageous to have a ratio closer to one as for gallium as this means that the two fluorescence signals *F*_*a*_ and *F*_*b*_ will have similar signal strengths. Indium has a much lower ratio because of the lower population in the upper state decreasing the fluorescence signal *F*_*b*_ which limits the indium measurements to above 1000 K. The temperature sensitivity of the two atoms is shown in Fig. 3b and is the derivative of the simulated ratio as a function of temperature, $\delta R_{\text{sim}} /\delta T$. At flame temperatures indium and gallium are seen to have similar sensitivity while at lower temperatures below 1000 K gallium has a much higher sensitivity than indium.

The concentration of the seeded species is evaluated from the signal acquired on the photodiode located after the flame. During a measurement the laser signal, $S_{m}$, after passing through the flame is recorded as well as the flame background luminescence, $S_{\text{flame bkg}}$, similar to the blue line in Fig. 1b. A reference laser signal, $S_{\text{no flame}}$, without absorption is acquired when the flame is turned off. A measurement of the absorption is, thus, calculated from the following expression.

$${\text{abs}} = 1 - \frac{{S_{m} - S_{\text{flame bkg}} }}{{S_{\text{no flame}} - S_{\text{bkg}} }}$$

Here $S_{\text{bkg}}$ is the background signal without both flame and laser. The reference photodiode located before the flame may be used to compensate the measurement should the laser power vary between the two measurements, $S_{m}$ and $S_{\text{no flame}}$. From the absorption measurement, the concentration can be derived according to Beer–Lambert’s law where the absorption cross section is inferred by the line-shape overlap function, acquired from the excitation scan, and the well-known Einstein coefficients.

## Results and discussion

In the following section, the experimental results are presented. Firstly, the distribution and concentration of indium and gallium atoms in the flame is investigated. Secondly, excitation scans of the four absorption lines, two for each atomic species, necessary for the overlap function are presented. Lastly, TLAF temperature measurements at different equivalence ratios for the two atomic species are compared to CARS temperature measurements and the accuracy and precision of the TLAF technique are evaluated.

### Species distribution and concentration

**Fig. 4:**
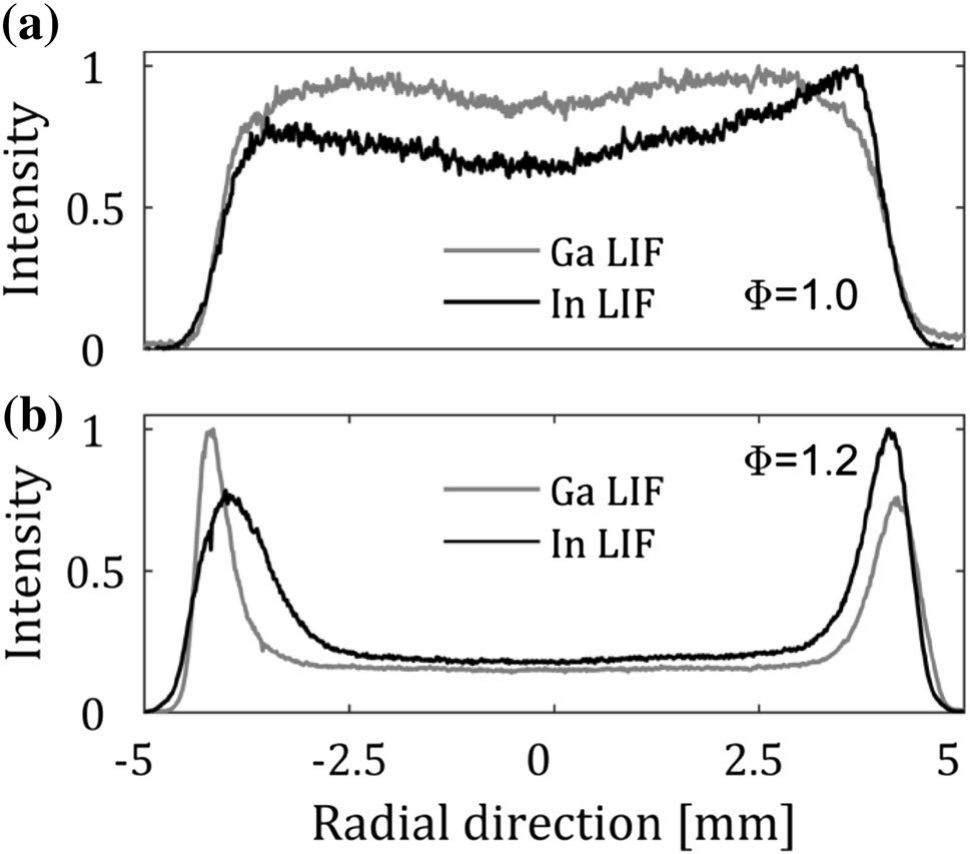
Radial profiles of the laser-induced fluorescence signals of indium and gallium for two equivalence ratios 1.0 and 1.2. The measurements were conducted 7 mm above the outlet of the burner

The distribution of indium and gallium over the multi-jet burner was investigated from the laser-induced fluorescence (LIF) signals collected at varying equivalence ratios in the product zone 38 mm above the jet nozzles. For lean and stoichiometric flames, a flat LIF signal was observed across the burner as shown for the stoichiometric case in Fig. 4a. For the rich flames, a strong increase in the LIF signal is observed in the secondary reaction zone where the unburned fuel meets the surrounding air. The increase of indium atoms in the secondary reaction zone may be explained by the decomposition of indium species and radicals in this chemical reactive region. For example, TMIn/TMGa may not have decomposed completely in a fuel-rich flame in the first reaction zone or formation of atomic radicals such as InOH (radicals previously observed to form in combustion environments in [25]) may dissociate in the second reaction zone.

**Fig. 5:**
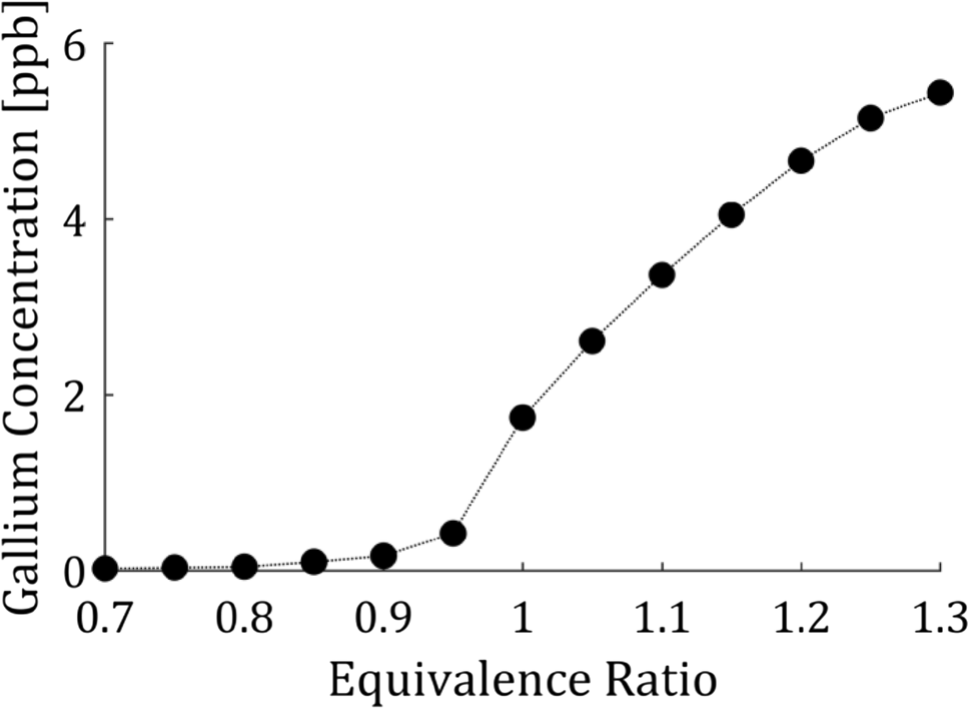
Concentration of gallium atoms in the flame for a constant seeding flow. The estimated seeding concentration of TMGa was 70 ppm giving at best a conversion to free atoms of 1‰ for the rich flames and even lower at lean flames. The concentration was measured 7 mm above the outlet of the burner. The estimated accuracy of the concentration measurements is 4% and the error bars within the circles

The concentration of gallium atoms in the seeded flame was investigated in relation to the equivalence ratio by keeping the seeding and total flow through the burner (33 SLM/min) constant and varying the equivalence ratio between 0.7 and 1.3. The seeding concentration of TMGa was determined to be 70 ppm derived from the flow through the bubbler, the vapor pressure of TMGa and the temperature of the bubbler (− 12 ºC) assuming that the residence time of the gas through the bubbler was long enough to saturate the carrier gas. The measured concentration of gallium atoms as a function of equivalence ratio is presented in Fig. 5 and it is observed that the concentration of gallium in the flame is at ppb levels. The errors in the quantification of the concentration measurements were estimated theoretically. The main errors and their corresponding effect on the temperature are (a) A line-shape overlap error of at most 0.0005 nm yielding concentration error of 2%, (b) inaccurately known constants, e.g. Einstein coefficient, of 2% resulting in a corresponding temperature error of 2% and (c) error in the signal measured on the photodiode by 2% resulting in an a temperature error of 2.8%. Assuming all errors are normally distributed the total error of the concentration measurements is 4%.

The discrepancy between the seeded concentration of TMGa and the concentration of gallium atoms may have several reasons. The largest loss is most probably the deposition of the seeded trimethyl compound on the surface of the gas lines and in the burner reducing the seeded concentration. Another reason may be that the TMGa is not completely converted into gallium atoms and that the seeded species reacts in the flame to form radicals such as GaOH and GaH as previously observed for indium flames [25]. The decline in atomic concentration observed when the flame becomes more and more fuel lean may be explained by oxidation of the atoms with, e.g., oxygen or hydroxyl radicals. Even though the conversion efficiency of the trimethyl compound is below 1‰, the signal is sufficiently strong for accurate temperature measurements and a higher seeding concentration would induce too much absorption.

### Excitation spectra

**Fig. 6:**
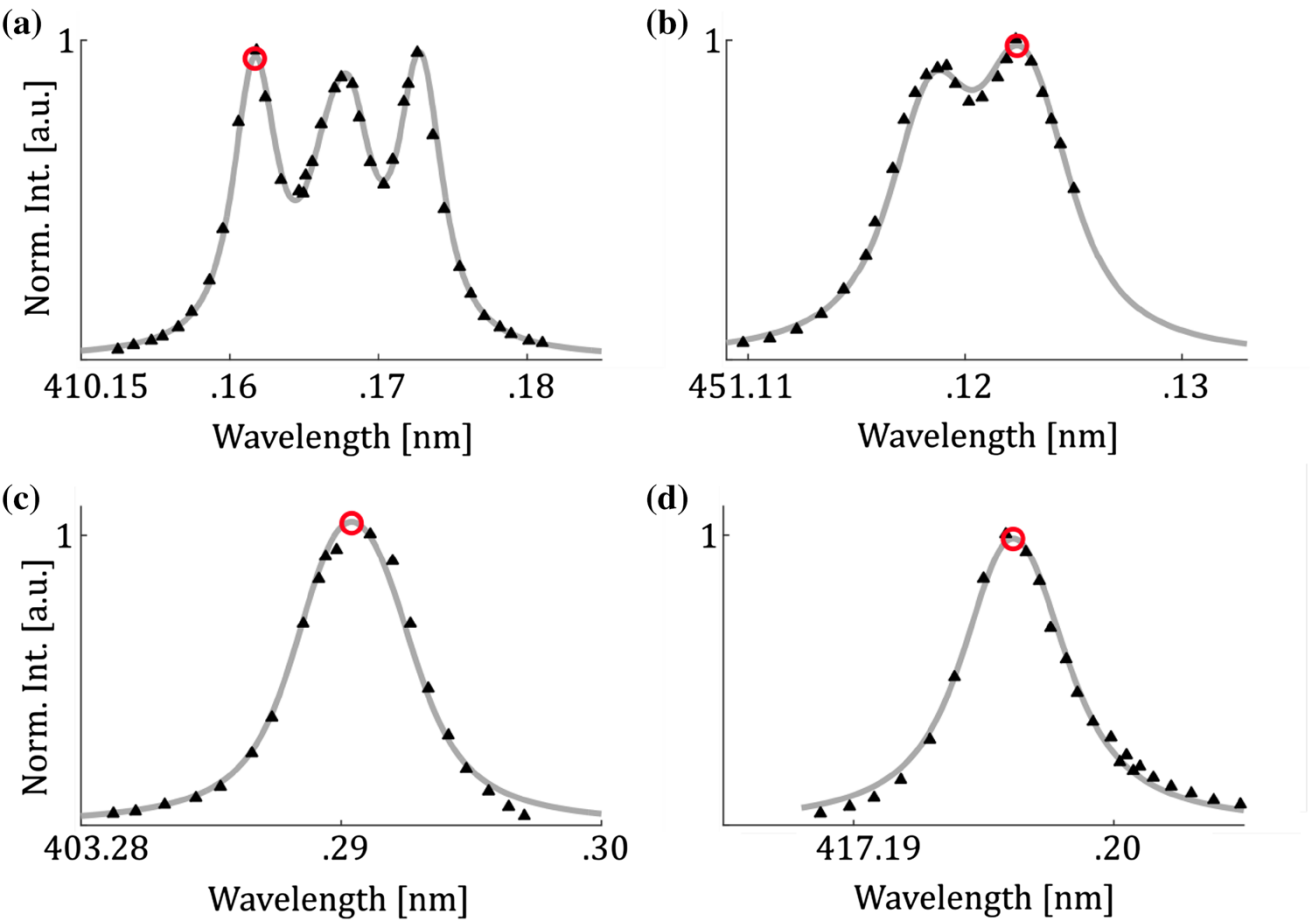
Excitation scans of the transition used in indium and gallium. The black triangles are measurement points and the gray line is a least-square fit to the data. The red circle marks the position of the laser during a thermometry measurement. **a** Gallium 4^2^P_1/2_ → 5^2^S_1/2_, **b** gallium 4^2^P_3/2_ → 5^2^S_1/2_, **c** indium 5^2^P_1/2_ → 6^2^S_1/2_, **d** indium 5^2^P_3/2_ → 6^2^S_1/2._ The excitation scans were conducted 7 mm above the outlet of the burner in a flame with equivalence ratio 1.0 and a corresponding temperature of 1950 K

The presented thermometric technique requires, as previously mentioned, knowledge of the overlap between laser frequency and absorption profile to provide accurate temperature measurements. This information was acquired from excitation scans of the four involved transitions, two for gallium 4^2^P_1/2_ → 5^2^S_1/2_ and 4^2^P_3/2_ → 5^2^S_1/2_ and two for indium 5^2^P_1/2_ → 6^2^S_1/2_ and 5^2^P_3/2_ → 6^2^S_1/2_. Excitation scans of the two transitions of gallium are shown in Fig. 6a, b and the two for indium are shown in Fig. 6c, d. All the excitation scans were conducted in a flame with equivalence ratio 1. Together with the excitation scans, least-square fits of the well-known hyperfine structures of the four transitions are presented as the gray lines, which agree well with the experimental data. The red circles in Fig. 6 mark the absorption peak and the wavelength the lasers were tuned to during the temperature measurements.

The hyperfine levels of gallium are more closely spaced as seen in Table 1 and results in the more homogenous absorption profiles compared to indium. In measurements with, e.g., dye lasers, common for instantaneous temperature measurements, it might be beneficial to have a narrower absorption profile like gallium to cover the complete line profile of the excited species where it is often assumed that the laser line width is much wider than the absorption profile. In one-line atomic fluorescence (OLAF), indium is better suited as the depths of the troughs have been shown to be temperature sensitive [29] whereas the gallium absorption profile does not have any trough structure. In the case of single-mode lasers with very narrow laser linewidths, a narrower absorption line will increase the signal strength with the downside of experiencing a higher self-absorption.

**Table 1:** The probed transitions of gallium and indium and information of the hyperfine structure used to simulate the absorption profile

Transition	Wavelength (nm)	Einstein A coefficient (10^8^ s^−1^)	Detection Filter (nm)	Filter peak transmittance (%)	Hyperfine splitting (GHz)	Relative intensities
Ga 4^2^P_1/2_ → - 5^2^S_1/2_	403	0.485	417 ± 5	50	0	1.0000
					2.138	0.3636
					2.678	0.6667
					4.816	1.0000
Ga 4^2^P_3/2_ → - 5^2^S_1/2_	417	0.945	417 ± 5	50	0	0.3571
					0.319	0.3571
					0.447	0.1429
					2.138	1.0000
					2.457	0.3571
					2.585	0.0714
In 5^2^P_1/2_ → - 6^2^S_1/2_	410	0.50	450 ± 5	50	0	1.0000
					8.428	0.3636
					11.422	0.6667
					19.810	1.0000
In 5^2^P_3/2_ → - 6^2^S_1/2_	451	0.89	450 ± 5	50	0	0.3385
					1.084	0.5077
					1.753	0.5385
					6.650	1.0000
					8.403	0.5077
					9.5200	0.1846

Gallium hyperfine splittings from [26] and indium hyperfine splittings from [27]. Relative intensities calculated from the Clebsch–Gordan coefficients

### Burner measurements

**Fig. 7:**
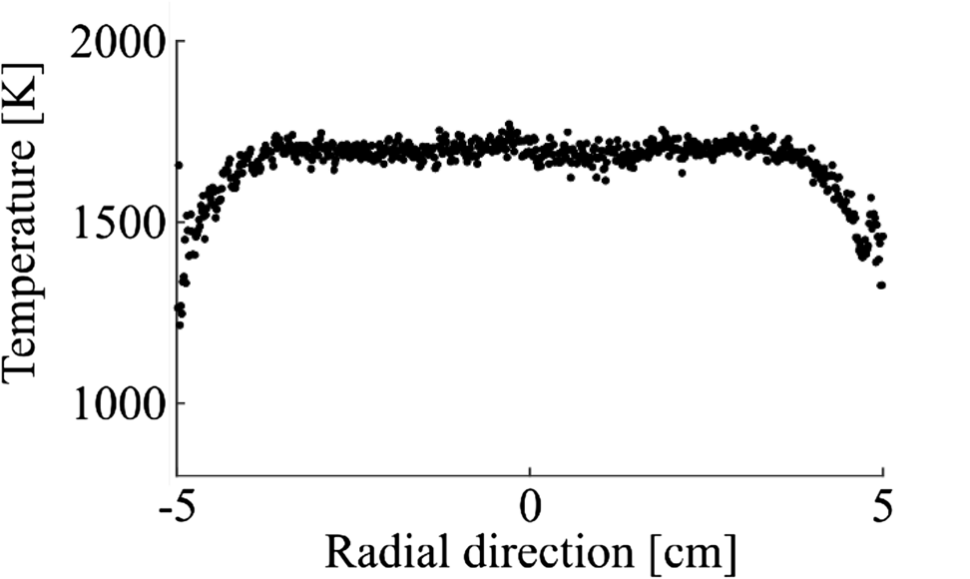
The radial temperature profile in the burner for an equivalence ratio of 1.1 measured with indium TLAF 7 mm above the burner outlet. A flat temperature profile is observed between − 4 and 4 cm

Temperature measurements in a reactive flow were conducted in a multi-jet burner [23] with the equivalence ratio varying from 0.7 to 1.3 allowing for temperatures between 1500 and 2000 K to be measured. The temperature measurements were conducted along a line in the center of the burner 7 mm above the outlet of the burner as shown in Fig. 1c. The temperature profile for the equivalence ratio 1.1 is presented in Fig. 7 and a flat temperature profile is observed between − 4 and 4 cm.

**Fig. 8:**
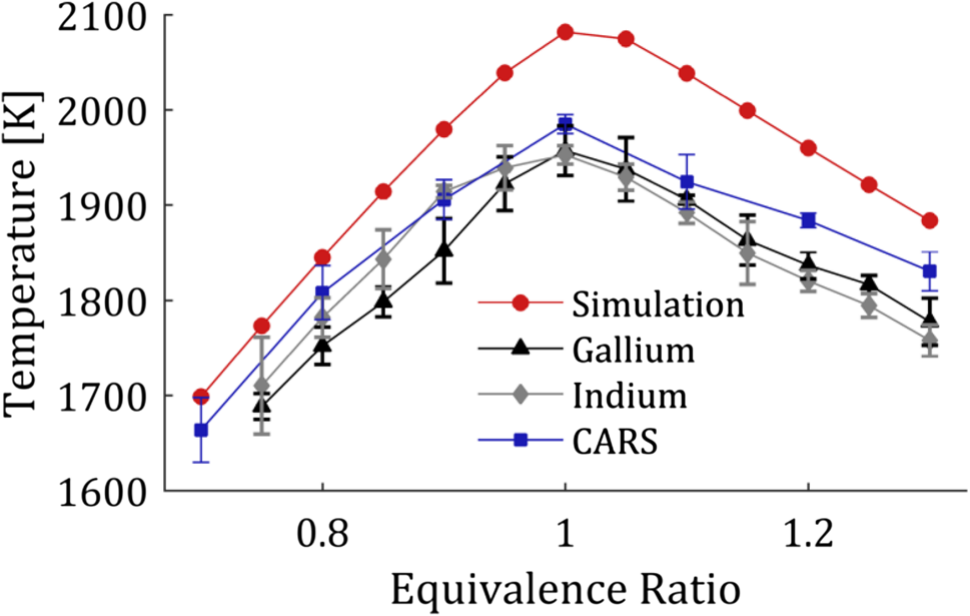
Temperature measured in the multi-jet burner as a function of equivalence ratio. Red dots: ChemKin simulation, blue squares: CARS temperature measurement, black triangles: TLAF Indium, gray diamonds: TLAF Gallium. Each measurement point is the average of three individual measurements and the error bars are the standard deviation these three measurements

An average temperature, extracted from the region in the flame with a flat temperature profile, − 4 to 4 cm, was calculated for both indium and gallium at the different equivalence ratios and is presented in Fig. 8. Each temperature point in Fig. 8 is an average of three separate measurements and the presented error bar is the standard deviation of these three measurements. The TLAF temperature measurements of indium and gallium are seen to agree well with each other. Temperatures were simulated using the GRI-Mech 3.0 [30] in ChemKin under the assumption of adiabatic conditions and are presented as the red dots in Fig. 8. The relatively higher simulated temperatures compared to the experimental results are due to the omission of heat losses in the simulation. However, the temperature trend for the experimental measurements compares well to the simulation. The standard deviation of the average temperature measurements is 1.2% for both indium and gallium, thus showing the repeatability of the technique is acceptable.

Rotational Coherent anti-Stokes Raman Spectroscopy (CARS) measurements [31] were conducted at the same height in the center of the burner to compare the TLAF measurements to a well-established thermometric technique. The evaluated temperature for each equivalence ratio shown in Fig. 8 is the average of three different CARS measurements. Since air was used as the oxidant, the rotational CARS spectra were dominated by lines from nitrogen, hence nitrogen thermometry was applied for the evaluation [32]. In the experiment, a dual broadband rotational CARS setup with a BOXCARS phase-matching scheme was used. To avoid any influence from smeared vibrational CARS originating from the C–H bonds in the fuel, a different spectral domain centered at 690 nm was chosen for the broadband pump and Stokes, rather than the commonly used centered at 630 nm [33]. For improved accuracy, the line-broadening coefficients on nitrogen from H_2_O, CO, H_2_ and N_2_ were included in the evaluation [2]. The TLAF measurements compare well with the CARS measurements showing the validity of the developed thermometric technique.

**Fig. 9:**
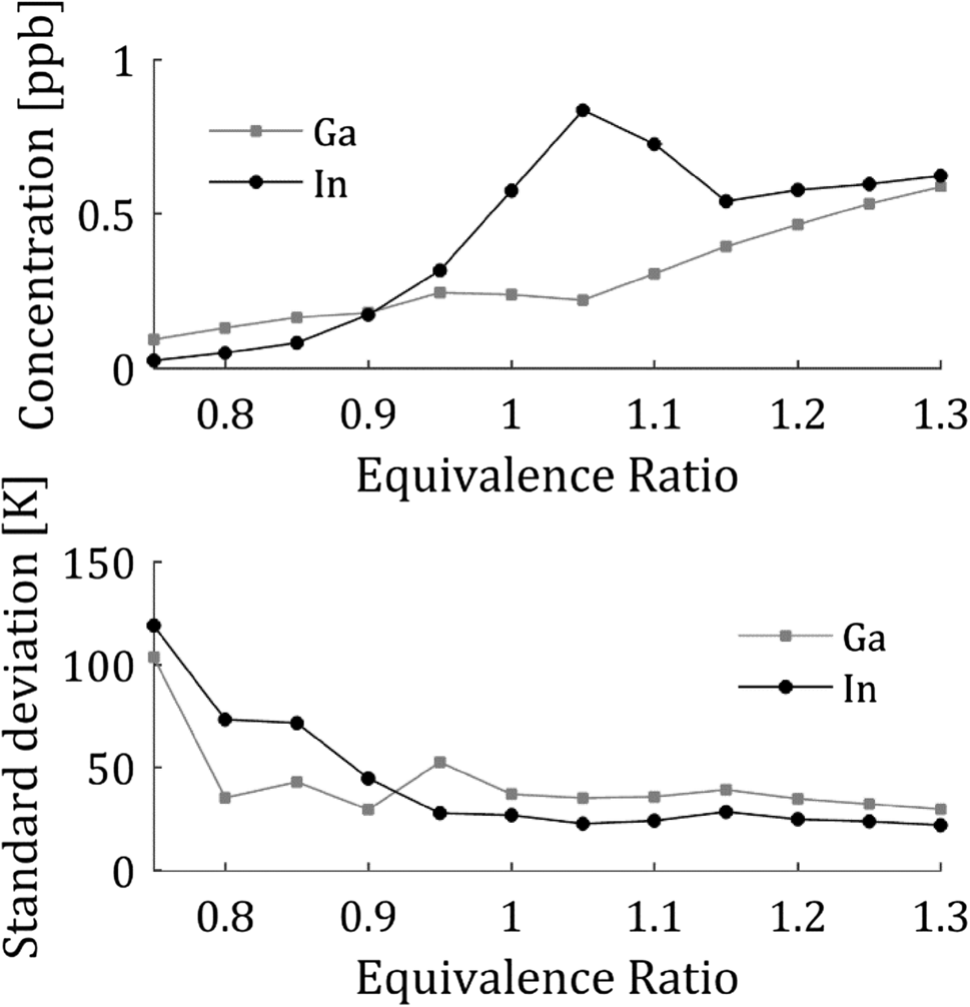
**a** The average concentration of the seeded atomic species for the measurements presented in Fig. 8. The seeding concentration was changed for each equivalence ratio to achieve acceptable signal levels while still without severe absorption. The estimated accuracy of the concentration measurements is 4% and the error bars are within the circles. **b** The standard deviation of the temperatures in the flat temperature region of the flame, see Fig. 7, for the different equivalence ratios. The standard deviation is seen to increase at leaner flames where the concentration and thus the signal is decreasing

The fluorescence signal, for the temperature measurements presented in Fig. 8, was optimized for the different equivalence ratios by adjusting the seeding concentration to maximize the fluorescence signal while still keeping the amount of absorption acceptable. In the current detection configuration, the self-absorption is not an issue as the detected signal is fluorescence to the same level independent on the excitation wavelength, i.e., both fluorescence signals will experience the same self-absorption. However, the absorption is a problem as the two excited levels have different absorption cross sections and is, thus, absorbed different amounts causing the ratio of the laser power, $I_{12} /I_{02}$, to change along the line of absorption, thus introducing a bias in the measurement. The average concentration for each equivalence ratio is presented in Fig. 9a and it is observed that the concentration is low for fuel lean flames even though the seeding concentration was increased to counter-act the effect of oxidation as observed in Fig. 5. A higher concentration for the fuel lean flames would increase the SNR and precision (discussed below); however, the seeding system limited the total seeded concentration to the amount presented here.

The accuracy and precision are important figures of merit for any thermometric technique. The performance of the developed TLAF technique is determined by a combination of experiments and theoretical estimations. The precision of the technique is affected by the signal-to-noise ratio of the experimentally determined fluorescence ratio and the temperature sensitivity of the probed atomic species. The SNR of the fluorescence ratio can be optimized by increasing the detected signal, e.g. by increasing the seeding concentration, higher camera gain, longer camera exposure and optimizing the collection optics. These parameters are all individual to the measurement configuration and will vary depending on the setup. The precision of the technique can be estimated from the standard deviation in a region of the flame where the temperature is expected to be homogenous, e.g., the plateau presented in Fig. 7. The precision of the measurements presented in Fig. 8 for the different equivalence ratios are shown in Fig. 9b. The precision is in this case mainly limited by the fluorescence signal where a decrease in signal results in a larger error in the precision. For the optimal case, e.g., for equivalence ratio 1.1 presented in Fig. 7 the precision was 1.1% of the measured temperature. The technique has a good precision making it suitable for detecting small changes in temperatures using a 2D setup.

After a certain signal level (in this case occurring around *Φ* = 1.0), the precision is flattening out and the precision is limited by the energy gap of the probed species where a larger gap gives better precision [12]. However, there is a trade-off of between how large the energy gap can be with the signal level of the upper ground state. Gallium has sufficient population of the upper ground state even at room temperature, assuming free gallium atoms could be produced at this temperature, while the laser-induced fluorescence signal of indium suffers below 1000 K due to the low population of the upper ground state.

There are several parameters that affect the accuracy of the presented TLAF technique. The overlap between the laser and absorption profile has to be explicitly known and this is the main parameter affecting the temperature accuracy. The overlap function is dependent on, (a) the simulation of the line profile based on the excitation scans, (b) small wavelength drifts and (c) the temperature dependence on the pressure shift. The maximum drift in laser wavelength that was observed in a measurement was at most 0.2 pm. The wavelength dependence of the accuracy was experimentally investigated by changing the wavelength of one of the lasers in steps of 0.1 pm around the peak of the absorption profile. It was found that a change in 0.2 pm introduced an error of 2%.

Although all experiments were conducted in atmospheric conditions there is a slight pressure shift due to the change in gas density with temperature. The pressure shift of the 5^2^P_1/2_ → 6^2^S_1/2_ line of indium with nitrogen is reported by Eberz et al. [34] and even if there are other species present in the flue gas of the burner nitrogen is the major collision partner and can thus be used to estimate the shift. There is no data reported for the three remaining lines but as gallium is homologous to indium one can assume the pressure shift of the lines is in the same order. The pressure shift only affect measurements differing in temperature from which the excitation scans were conducted (in this case *Φ* = 1.0 and *T* = 1940 K.) The pressure shift is approximately 0.8 pm for a temperature change of 500 K which compared to the wavelength drift is four times larger. However, all lines are shifted simultaneously and thereby mitigating some of the temperature error that would occur of only one of the lines was shifted. The pressure shift induced for a temperature difference of 200 K between the excitation scan and the measurement results in an error of 0.5% and the corresponding error for a 500 K difference is 1%.

The laser power calibration will also influence the accuracy of the technique. For the laser power calibration, the power meter has a specified absolute uncertainty of 2%. The error in the power measurement between the two wavelengths is liberally estimated to 2% resulting in a temperature error of at most 1.5%. The total error is calculated by assuming the errors are normally distributed and results in a total accuracy of 2.7%.

## Conclusion

A calibration-free diode laser-based thermometric technique based on two-line atomic fluorescence has been developed. The technique has been used to compare indium and gallium as temperature markers for TLAF. This is the first time gallium has been used in TLAF to the authors’ knowledge. The thermometric technique was estimated to have an accuracy of within 2.7% for averaged measurements and a precision of 1%. The temperature was measured in a laminar methane-air flame in a temperature range from 1600 to 2000 K at different equivalence ratios. Both the indium and gallium temperature measurements were in good agreement with CARS measurements. No difference in accuracy or precision for the two atomic species was observed in experiments at flame temperatures. The concentration of both atomic species was seen to decrease for leaner flames due to oxidation.

Gallium has been shown to be a good candidate as temperature marker for TLAF and has the potential to be able to provide temperature measurements at lower temperatures than indium, important for low temperature chemical reactions, assuming gallium atoms can be generated at these temperatures.
